# Updates in Small Interfering RNA for the Treatment of Dyslipidemias

**DOI:** 10.1007/s11883-023-01156-5

**Published:** 2023-10-04

**Authors:** S. Carugo, C. R. Sirtori, G. Gelpi, A. Corsini, L. Tokgozoglu, M. Ruscica

**Affiliations:** 1 Department of Clinical Sciences and Community Health, Dyspnea Lab, Università degli Studi di Milano, Milan, Italy; 2 Department of Cardio-Thoracic-Vascular Diseases - Foundation IRCCS Cà Granda Ospedale Maggiore Policlinico, Milan, Italy; 3 Department of Pharmacological and Biomolecular Sciences, University of Milan, Milan, Italy; 4 Department of Cardiology, Hacettepe University Faculty of Medicine, Ankara, Turkey

**Keywords:** ARO-ANG3, ARO-APOC3, Inclisiran, Lipid-lowering therapy, Olpasiran

## Abstract

**Purpose of Review:**

Atherosclerotic cardiovascular disease (ASCVD) is still the leading cause of death worldwide. Despite excellent pharmacological approaches, clinical registries consistently show that many people with dyslipidemia do not achieve optimal management, and many of them are treated with low-intensity lipid-lowering therapies. Beyond the well-known association between low-density lipoprotein cholesterol (LDL-C) and cardiovascular prevention, the atherogenicity of lipoprotein(a) and the impact of triglyceride (TG)-rich lipoproteins cannot be overlooked. Within this landscape, the use of RNA-based therapies can help the treatment of difficult to target lipid disorders.

**Recent Findings:**

The safety and efficacy of LDL-C lowering with the siRNA inclisiran has been documented in the open-label ORION-3 trial, with a follow-up of 4 years. While the outcome trial is pending, a pooled analysis of ORION-9, ORION-10, and ORION-11 has shown the potential of inclisiran to reduce composite major adverse cardiovascular events. Concerning lipoprotein(a), data of OCEAN(a)-DOSE trial with olpasiran show a dose-dependent drop in lipoprotein(a) levels with an optimal pharmacodynamic profile when administered every 12 weeks. Concerning TG lowering, although ARO-APOC3 and ARO-ANG3 are effective to lower apolipoprotein(apo)C-III and angiopoietin-like 3 (ANGPTL3) levels, these drugs are still in their infancy.

**Summary:**

In the era moving toward a personalized risk management, the use of siRNA represents a blossoming armamentarium to tackle dyslipidaemias for ASCVD risk reduction.

## Introduction

Atherosclerotic cardiovascular disease (ASCVD) is still the leading cause of death worldwide despite excellent pharmacological approaches and revascularizations [1, 2]. As strongly supported by epidemiologic and interventional studies as well as by genetic evidence, elevated levels of low-density lipoprotein cholesterol (LDL-C) are considered a major causal factor for ASCVD [3]. Thus, keeping LDL-C concentrations low to minimize the rate of progression of atherosclerotic plaques is a major strategy to reduce the risk of events [4]. The achieved lowering of LDL-C was directly associated with a reduced incidence of major ASCVD events [5]. This benefit is maintained up to very low levels of LDL-C. In fact, a threshold level has not yet been identified [6]. Despite excellent pharmacological approaches [7], a gap between clinical guidelines and clinical practice still stands. The DA VINCI [8] and the SANTORINI [9] studies showed that, among European patients at high and very high-risk for ASCVD, only a small percentage (roughly between 20 and 33%) reach present-day LDL-C targets.

Beyond LDL-C alone and familial hypercholesterolemia, the atherogenicity of lipoprotein(a) and triglyceride (TG)-rich lipoproteins (TRL) cannot be overlooked. Specifically related to lipoprotein(a), there exists a continuous relationship between plasma concentrations and risk for endpoints of ASCVD [10, 11]. On an equimolar basis, lipoprotein(a) is more atherogenic than LDL because it carries all the proatherogenic components of LDL in addition to apo(a), that binds phosphocholine containing oxidized phospholipids [12]. Within the context of residual risk, robust and growing evidence from epidemiologic and genetic studies suggest that TRL and their remnants are causally related to the risk of ASCVD [13–15].

Considering that targeted delivery of nucleic acid–based therapies has progressed substantially in recent years [16], the present review will investigate therapeutic approaches targeting ribonucleic acids, underscoring the versatility of oligonucleotide therapeutic agents and their potential to target previously undruggable pathways [17]. The hybridization of each oligonucleotide drug to the target leads to the activation of endogenous enzymes, thus resulting in cleavage of the targeted mRNA at the site of hybridization [18]. In particular, we focused on double-stranded small inhibiting RNAs (siRNA) that target the mRNA of proprotein convertase subtilisin/kexin type 9 (*PCSK9*) gene to lower LDL-C, of *LPA* gene to lower lipoprotein(a), and of apolipoprotein C3 (*APOC3*) and angiopoietin-like 3 (*ANGPTL3*) genes to reduce triglycerides. Although the use of monoclonal antibodies is highly effective, as in the case of PCSK9 antagonism, for proteins without enzyme activity (e.g., lipoprotein(a) or apolipoprotein C-III), this approach would require the use of a large mass of antibodies with the risk of generating large amounts of immune complexes [19].

## Nucleic Acid–Based Approaches—Small Interfering RNA

**Fig. 1:**
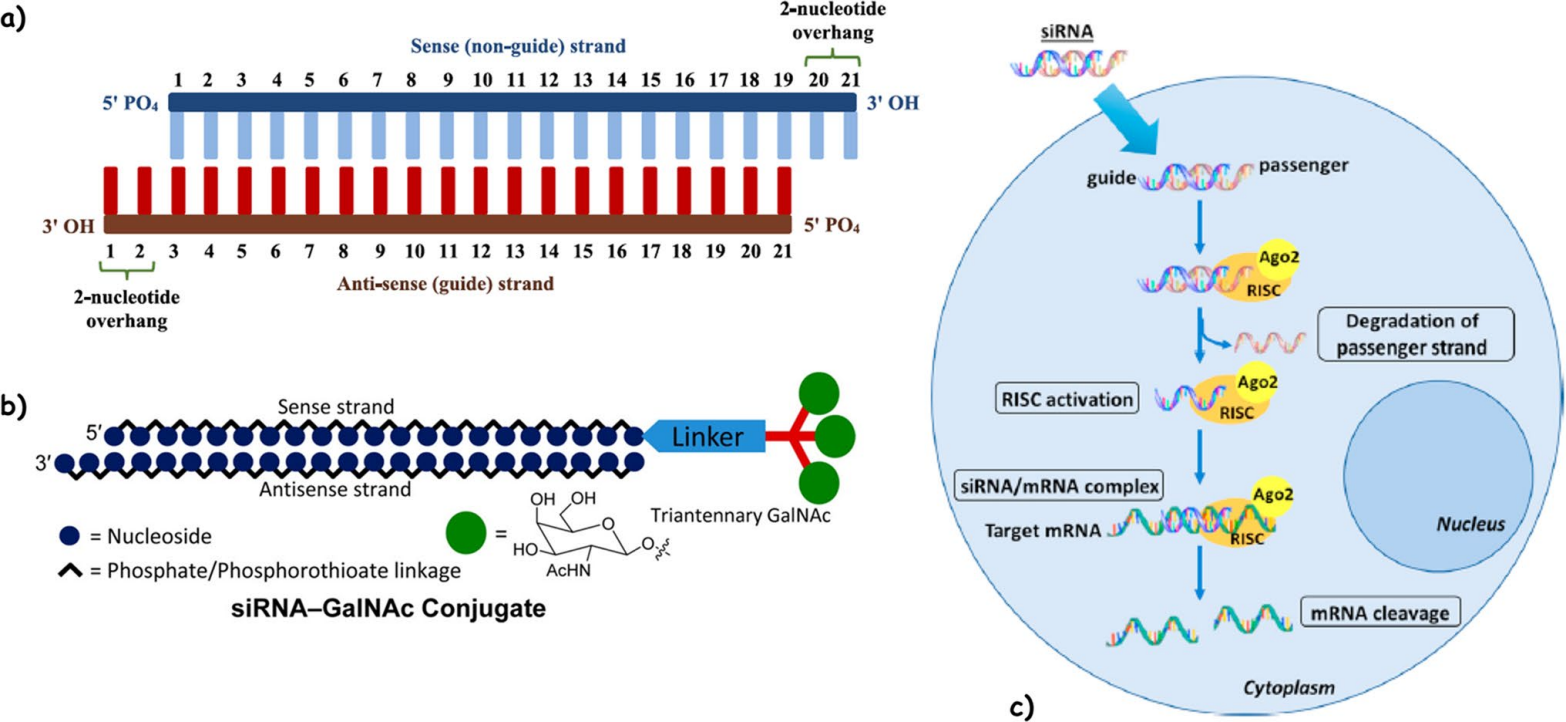
Small interfering RNA (siRNA) structure and mechanism. **a** siRNA structure; **b** siRNA conjugated with N-acetylgalactosamine (GalNac); **c** once in the cytoplasm the two strands of siRNA are separated with the guide loaded into the RISC and the passenger removed and degraded. When the complementary target mRNA has hybridized with part of the guide strand, an endonucleolytic cleavage of the mRNA is driven by a component of RISC, the Argonaute 2 (ago 2) protein. (Modified with permission from: [**Panel a**] John Wiley and Sons ©2022 [24]; [**Panel b**] American Chemical Society ©2014 [25]; and [**Panel c**] Elsevier ©2019 [26])

RNA interference is a naturally occurring molecular phenomenon with micro RNAs and some long noncoding RNAs exerting their function with high specificity by complementary base-pairing to their RNA targets [20]. The description of this process allowed Andrew Z. Fire and Craig C. Mello to be awarded the Nobel Prize in Physiology or Medicine in 2006. RNA interference is a conserved biological process allowing a mRNA to be destroyed in response to double-stranded RNA (dsRNA) [21]. The starting event for the RNA interference pathway is the cleavage of long dsRNA molecules into short small interfering RNA (siRNA) fragments, 21–23 bp in length, by a member of the ribonuclease (RNase) III family called DICER. Thus, synthetic siRNAs are aimed at silencing specific target genes by mimicking the structure of DICER products. Synthetic siRNAs are composed of two strands, the guide (anti-sense strand) containing the information for target-gene recognition and the passenger (sense strand) supporting the geometry required to be loaded into the RISC (RNA-induced silencing complex) [22]. Once in the cytoplasm, the two strands are separated with the guide loaded into the RISC and the passenger removed and degraded. RISC uses the guide RNA to find complementary mRNA sequences via Watson-Crick base pairing. When the complementary target-mRNA has hybridized with part of the guide strand, an endonucleolytic cleavage of the mRNA is driven by a component of RISC, the Argonaute 2 (Ago2) protein [23]. Because Ago2 is primarily localized to the cytoplasm, siRNAs effectively target cytoplasmic RNAs (Fig. 1).

In spite of the promising efficacy of siRNAs, to overcome some setbacks, many chemical modifications have been introduced to increase the affinity per nucleotide unit for the cognate sequence and/or to enhance resistance to nucleases, the enzymes that degrade these drugs [27]. To improve stability, the most widely used modifications pertain to (i) substitution of the 2′ position of the sugar ring which includes 2′-O-methyl,2′-fluoro, or 2′-methoxyethyl [28], and (ii) the introduction of phosphorothioate modifications in place of the two terminal phosphodiester linkages of each strand of the siRNA [29]. However, for chemical modifications and delivery modalities, we suggest specific reviews on this topic [24, 27, 30].

Finally, encapsulating siRNAs into vesicles or by the conjugation of moieties with high binding capacity for receptors can effectively avoid renal clearance and can guarantee the deliver to the desired tissues or cells [31]. A major breakthrough in the field was the conjugation of N-acetylgalactosamine (GalNAc) moieties to siRNA. This enabled the use of this chemical class of drugs for targets expressed in hepatocytes without the need for pro-inflammatory liposome formulations [25]. Overall, covalent attachment of a synthetic triantennary GalNAc ligand to chemically modified siRNA has enabled asialoglycoprotein (ASGPR)-mediated targeted delivery of therapeutically active siRNAs to hepatocytes [32]. ASGPR expression on hepatocytes is abundant, with 200 to 500,000 copies per cell. Upon uptake of the GalNAc conjugates by the ASGPR, the GalNAc moiety is rapidly cleaved from the ASGPR after which the receptor recycles back to the cell membrane. Since the oligonucleotide escapes the endo-lysosomal compartment leading to a sort of endosomal storage, this explains the surprisingly sustained silencing activity (up to 18 months) [33].

## Inclisiran to Lower PCSK9

In the era of monoclonal antibodies against PCSK9 that dramatically reduce LDL-C when used alone or in combination with statins, a new RNA-based approach has been approved by the FDA (in 2021) and EMA (in 2020) as a lipid-lowering agent.

However, it is worth acknowledging that already in 2008, a small interfering RNA, formulated in a lipidoid nanoparticle (LNP), was able to target hepatic PCSK9 leading to lower plasma cholesterol levels in rodents and LDL-C in nonhuman primates. In transgenic mice expressing human PCSK9, this siRNA silenced the human PCSK9 transcript by >70% and significantly reduced PCSK9 plasma protein levels [34]. In an early randomized phase 1 trial, ALN-PCS treatment (0.400 mg/kg) yielded 70% and 40% reductions in plasma PCSK9 and LDL-C, respectively, compared to placebo [35]. However, a major setback was the formulation of ALN-PCS, flawed for clinical use because of inadequate duration of effects. This study was followed by a phase 1 trial with a long-acting RNA interference, known as inclisiran (ALN-PCSsc) [36]. In healthy volunteers, inclisiran at doses of 300 mg or more (in single or multiple dose regimens) reduced PCSK9 levels and LDL-C for at least 6 months. These results were the ground for the development of the ORION program testing the safety and efficacy of inclisiran in different clinical settings of dyslipidemias (Table 1). The positive results of the first phase 3 clinical trials of the ORION program have led the FDA and EMA to approve, in 2021, the use of inclisiran as a lipid-lowering agent in patients with ASCVD and heterozygous familial hypercholesterolemia (HeFH). In early 2023, the FDA expanded indications to treat adults with high LDL-C and who are at increased risk of heart disease [50].

**Table 1:** List of trials evaluating safety and efficacy of inclisiran

	Trial identifier	Population	Primary endpoint	Main findings
ORION-1	NCT02597127	Participants with cardiovascular disease and high cholesterol	% change in LDL-C	The 2-dose 300-mg regimen produced the highest proportion of responders at day 360 and the greatest mean reduction in LDL-C over 1 year [37, 38]
ORION-2	NCT02963311	Homozygous familial hypercholesterolemia	% change in LDL-C	Review of data from these four participants provided sufficient data to justify a long-term phase 3 trial (ORION-5) [39]
ORION-3	NCT03060577	4-year follow-up of ORION-1	% change in LDL-C	The 4-year averaged mean reduction of LDL-C cholesterol was 44.2% (*95% CI*: 47.1–41.4) [40••]
ORION-4	NCT03705234	ASCVD	Major adverse cardiac events	Estimated study completion (July 2026)
ORION-5	NCT03851705	Homozygous familial hypercholesterolemia	% change in LDL-C	Ongoing
ORION-6	-	Patients with mild (Child-Pugh A) or moderate (Child-Pugh B) hepatic impairment and with normal hepatic function	Pharmacokinetic and pharmacodynamic profile	The exposure of inclisiran increased by up to two-fold in patients with moderate hepatic impairment. Pharmacodynamic effects remained relatively unchanged [41]
ORION-7	NCT03159416	Comparing patients with renal impairment and normal renal function	Pharmacokinetic parameters	Dose adjustments of inclisiran are not required in these patients [42]
ORION-8	NCT03814187	Patients with established ASCVD or very high risk of ASCVD (familial hypercholesterolemia or type 2 diabetes) and elevated LDL-C. It is open-label, active comparator extension trial of the ORION-3, ORION-9, ORION-10, and ORION-11 trials	Proportion of patients reaching LDL-C goals (<70 mg/dL or <100 mg/dL) based on the ASCVD risk level by day 1080	Among 3274 patients, the longest exposure to inclisiran was 6.84 years with a mean exposure of 3.7 years 79.4% of ASCVD patients reached LDL-C < 70 mg/dL and 74.3% of ASCVD risk equivalent reached LDL-C < 100 mg/dL LDL-C was reduced from baseline by 51% (*95% CI* 52.2–49.9) in ASCVD patients and by 42.4% (*95% CI* 45–39.9) in ASCVD risk equivalent patients [43]
ORION-9	NCT03397121	Heterozygous familial hypercholesterolemia	% change in LDL-C	LDL-C was reduced by 47.9% at day 510 [44]
ORION-10	NCT03399370	ASCVD and elevated LDL-C	Absolute change in LDL-C	Reductions in LDL-C levels of approximately 50% [45]
ORION-11	NCT03400800	ASCVD or ASCVD-risk equivalents and elevated LDL-C	Absolute change in LDL-C	Reduction in LDL-C levels of approximately 50% [45, 46]
ORION-12	Registration of this phase I trial was not required as it is not an applicable clinical trial according to current US regulatory guidance	48 healthy volunteers	To determine whether a supratherapeutic dose of inclisiran had any effect on cardiac repolarization	Inclisiran, at a supratherapeutic dose (900 mg), did not show a clinically significant effect on the QT interval [47]
ORION-13	NCT04659863	Adolescents with homozygous familial hypercholesterolemia	% change in LDL-C	Not available
ORION-14	NCT04774003	Chinese patients with hypercholesterolemia (LDL-C ≥100 mg/dL) who were on maximally tolerated statin	Safety, pharmacokinetics and LDL-C lowering effects	Single dose of 100 and 300 mg, significantly reduced LDL-C levels up to day 90 [48]
ORION-16	NCT04652726	Adolescents with heterozygous familial hypercholesterolemia	% change in LDL-C	Not available
ORION-18	NCT04765657	Asian Participants with ASCVD or ASCVD high risk and elevated LDL-C	% change in LDL-C	Not available
VICTORION-2 PREVENT	NCT05030428	Participants with established cardiovascular disease	Reduction in the risk of 3-point major adverse cardiac events: a composite of CV death, non-fatal myocardial infarction, and non-fatal ischemic stroke	Estimated study completion 2027
VICTORION- INCEPTION	NCT04873934	Patients with a recent acute coronary syndrome	% change in LDL-C	Not available

*ASCVD*, atherosclerotic cardiovascular disease; *CV*, cardiovascular; *LDL*, low-density lipoprotein cholesterol. *CI*, confidence interval. (Modified with permission from: Springer Nature via http://creativecommons.org/licenses/by/4.0/) [49]

Considering that the results of the ORION trials have been extensively described elsewhere [46, 51–53], hereto we discuss the most recent pooled analysis of ORION-9, ORION-10, and ORION-11 trials [54] along with the results of ORION-3, the 4-year open-label extension of the 1-year ORION-1 trial [40••]. In the ORION-1 trial, six different inclisiran dosing regimens were tested in patients at high risk for cardiovascular disease who had elevated LDL-C levels. At day 180, the group given inclisiran saw a mean absolute reduction in LDL-C levels of 64.2 ± 20.7 mg/dL [37]. However, this trial exposed patients to only four drug injections. Thus, to assess the efficacy of LDL-C lowering at day 210 and the durability of this effect over a 4-year follow-up, the ORION-3 open label extension study was run. Patients who were given placebo in the ORION-1 trial received evolocumab (twice a month) up to 1 year and then were transitioned to inclisiran. Those already on inclisiran in ORION-1 trial received the first dose at day 360 and thereafter at day 450 and then every 6 months until day 1350. This regimen has led to an overall LDL-C reduction of 44.2%, allowing 79% of patients to reach LDL-C levels < 70 mg/dL and 62% to reach < 50 mg/dL. The levels of PCSK9 were reduced between 62.2% and 77.8% [40••]. However, in a real-world setting, a substantial interindividual variability of LDL-C reductions has been observed after the first and second administration of inclisiran [55]. In particular, in patients previously treated with PCSK9 mAbs, LDL-C reductions were less effective (roughly 24%) than in PCSK9-mAbs-naïve patients (roughly 40%) at 3 months. Within this context, it should be recall that PCSK9 inhibition, through monoclonal antibodies, enhances the secretion of PCSK9, an effect that contributes to the increased plasma PCSK9 levels in treated subjects [56].

Although data of ORION-3 have undoubtedly demonstrated the durability of LDL-C lowering mediated by inclisiran, we need to await the results of ORION-4 (NCT03705234) and VICTORION-2 PREVENT (NCT05030428) studies which are testing the hypothesis that inclisiran reduces major adverse cardiovascular events (MACE) in patients with clinical ASCVD or high ASCVD risk. In the meantime, a prespecified analysis of ORION-9, ORION-10, and ORION-11 showed the potential of inclisiran to reduce composite MACE by 26% (*OR*: 0.74; *95%CI* 0.58–0.94), but not fatal and non-fatal myocardial infarction (*OR*: 0.80; *95%CI* 0.50–1.27) or fatal and non-fatal stroke (*OR*: 0.86; *95%CI* 0.41–1.81) [54]. Further pooled post hoc analyses of these three studies further highlighted the superiority of inclisiran (given as a twice-yearly dosing) vs placebo to reduce LDL-C consistently in patients with polyvascular [57] and cerebrovascular diseases [58].

Inclisiran will be tested in adolescent diagnosed with both homozygous familial hypercholesterolemia (HoFH) (ORION-13) and HeFH (ORION-16). In both trials, the primary endpoint is the percentage change in LDL-C from baseline to day 330 (1 year). Secondary endpoints are the evaluation of other lipid parameters (e.g., non-HDL-C, VLDL-C, TG, apolipoprotein(apo)B, and lipoprotein(a)) along with the occurrence of treatment-emergent adverse events [59].

Another important open question in the field of cardiovascular diseases remains the utility of reducing PCSK9 levels soon after an acute coronary syndrome event [60]. To answer this question, the aim of the VICTORION-INCEPTION study (NCT04873934) is to evaluate the effectiveness of implementation of a systematic LDL-C management pathway including treatment with inclisiran in participants who have experienced a recent acute coronary syndrome and have an increased LDL-C (≥70 mg/dL) despite being treated with a statin [61].

### Safety

The safety data extrapolated from the ORION-9, ORION-10, and ORION-11 showed that treatment-emergent adverse events (TEAEs) leading to drug discontinuation were reported in 2.5% of patients given inclisiran and in 1.9% of patients receiving placebo. TEAEs at the injection site were 5% with inclisiran and 0.7% with placebo. These effects were predominantly mild, and none was severe or persistent. Although bronchites was 4.3% with inclisiran and 2.7% with placebo, with a risk ratio of 1.55 (*95%CI* 1.09–2.20), these cases were nearly all mild to moderate. Nasopharyngitis rates were similar between groups. Liver and renal functions were preserved in both arms of the trials. The worsening of glycemic control was similar between groups (inclisiran: 11.6% vs placebo: 11.4%) [51], reassuring that inhibition of PCSK9 should be of minimal concern [62]. Concerning serious TEAEs, e.g., number of deaths, these were 27 in both the inclisiran and the placebo group [51].

## Olpasiran and SLN360 to Reduce Lipoprotein(a)

The most polymorphic of lipoproteins, lipoprotein(a) is a hybrid lipoprotein composed of a LDL-like particle containing one molecule of apoB-100 covalently bound to apo(a), a glycoprotein characterized by repeats of an unusual “kringle” structure (remindful of a Scandinavian pastry) [63]. Although epidemiological, genome-wide association and Mendelian randomization studies have shown that high lipoprotein(a) levels are a risk factor for ASCVD [64], so far, there is a lack of clinical trials demonstrating that a selective reduction in elevated lipoprotein(a) reduces the incidence of cardiovascular diseases. Since lipoprotein(a) levels are mostly genetically determined (between 70% and 90%) and minimally influenced by diet and lifestyle [65], the major National Lipid Associations recommend the use of lipoprotein(a) for risk stratification [66–69].

Olpasiran (formerly AMG890) is a GalNAc-conjugated siRNA directed against the mRNA of the *LPA* gene. Olpasiran is modified with 2′-fluoro and 2′-methoxy substitutions and phosphorothioate internucleotide linkages at the termini to stabilize the duplex. It was initially tested in a dose-escalating (3, 9, 30, 75, or 225 mg) phase 1 trial (NCT03626662), enrolling participants with lipoprotein(a) concentrations, between 70 and 199 nmol/L and ≥ 200 nmol/L. The lipoprotein(a) levels were reduced in a dose-responsive manner with a maximum decrement from baseline ranging from −71% to −97%. The effects last months after a single dose [70]. These results set the stage for the design of the OCEAN(a)-DOSE trial (Olpasiran trials of Cardiovascular Events And lipoproteiN(a) reduction-DOSE finding study) [71] which was aimed at testing olpasiran in 281 individuals with median lipoprotein(a) levels of 260.3 nmol/L and a history of ASCVD (including established coronary heart disease, peripheral artery disease, or atherosclerotic cerebrovascular disease). These inclusion criteria were different from those of Lp(a)HORIZON trial focused on a population with a history of events, indicating a higher-risk phenotype. Lp(a)HORIZON recruited patient with evidence of myocardial infarction, ischemic stroke, or peripheral arterial disease (PAD) within 10 years of enrollment [72].

Concerning the OCEAN(a)-DOSE trial, the dose-regimen consisted of 10 mg or 75 mg or 225 mg every 12 weeks and 225 mg every 24 weeks. Lipoprotein(a) levels were reduced in a dose-dependent fashion with the optimal pharmacodynamic effects when the drug was administered every 12 weeks. Placebo-adjusted mean percent changes in lipoprotein(a) levels were −70.5% with the dose of 10 mg, −97.4% with the dose of 75 mg, −101.1% with the dose of 225 mg every 12 weeks, and −100.5% with the dose of 225 mg every 24 weeks [73]. Subjects on doses ≥75 mg (every 12 weeks) sustained roughly 40–50% placebo-adjusted reduction in lipoprotein(a) levels close to 1 year after the last dose [74]. The long-term clinical efficacy and safety of olpasiran will be evaluated in the OCEAN(a)-Outcomes trial (NCT05581303) recruiting participants with ASCVD and elevated lipoprotein(a) levels.

Among the clinical strategies targeting apo(a) production, another GalNAc conjugated siRNA, the SLN360 was tested [75, 76]. A phase 1 escalating-dose trial, enrolling adults with lipoprotein(a) concentrations ≥ 150 nmol/L at screening and no known clinically overt cardiovascular disease, was run. Over 150 days, lipoprotein(a) levels were reduced from baseline as follows: −10% (with placebo), −46% (with the dose of 30 mg), −86% (with the dose of 200 mg), −96% (with the dose of 300 mg), and −98% (with the dose of 600 mg). However, the safety review committee recommended extending follow-up of lipoprotein(a) levels for participants in the two highest dose groups (namely, 300 and 600 mg) from 150 days to 1 year.

### Safety

The incidence of adverse events leading to discontinuation was similar (roughly 2%) in patients receiving olpasiran or placebo. Specifically, no differences were found concerning new-onset or worsening diabetes mellitus or myalgias. Liver- and kidney-related adverse events as well as thrombocytopenia were infrequent with a similar percentage in the olpasiran and placebo groups. In total, 17% of patients allocated to olpasiran experienced an injection site reaction compared with 11% in placebo arm [73].

### ARO-APOC3 and ARO-ANG3 to Silence apoC-III and ANGPTL3

Although preclinical, epidemiologic, and genetic evidence has provided strong support for a causal association between TG, TRL, and TRL remnants, and increased risk of ASCVD [14], the results of the PROMINENT (The Pemafibrate to Reduce Cardiovascular Outcomes by Reducing Triglycerides in Patients with Diabetes) study cast doubts whether lowering TG levels per se would also lower the incidence of cardiovascular events [77]. This uncertainty has provided impetus for the development of innovative therapeutic strategies to lower TG, TRL, and TRL remnants for potential cardiovascular benefit. Within this frame, Mendelian randomization studies, focused on variants in the genes encoding to *APOC3* and *ANGPTL3*, *ANGPTL4*, and *ANGPTL8*, were highly informative. Carriers of loss-of-function mutations of *APOC3* were characterized by low levels of TG and had a reduced risk of myocardial infarction [78••, 79]. Individuals heterozygous for ANGPTL3 had 41% lower odds of ASCVD, whereas individuals with complete ANGPTL3 deficiency had also reduced odds of myocardial infarction with no evidence of coronary atherosclerotic plaques [80, 81].

### APOC3

Mainly secreted by the liver and to a lesser extent by the intestine [82], apoC-III is a key regulator of TRL metabolism through lipoprotein lipase-dependent and lipase-independent pathways [83]. ApoC-III reduces lipoprotein lipase activity; it inhibits the removal of TG-rich particles from the bloodstream; it promotes hepatic VLDL secretion into the blood; it inhibits the turnover of TRL primarily through a hepatic clearance mechanism mediated by the LDL receptor/LDL receptor–related protein 1 axis [84].

ARO-APOC3 is a double-stranded, hepatocyte-target RNA interference which specifically silences the *APOC3* mRNA with the aim of reducing TG levels. The safety and pharmacodynamics of ARO-APOC3 was tested in a phase 1 trial enrolling healthy adult volunteers and participants with severe hypertriglyceridemia (NCT03783377) [85]. ARO-APOC3 was given in a single-dose escalation design (10, 25, 50, or 100 mg) in healthy volunteers with TG >80 mg/dL at baseline. Mean maximum reduction from baseline in serum apoC-III levels ranged from 72% (at the dose of 10 mg) to 94% at the dose of 100 mg. This reduction was maintained through the end of study (at week 16), with mean reductions of 70% (at the dose of 25 mg) to 91% (at the dose of 100 mg). TG and very low-density lipoprotein cholesterol (VLDL-C) were reduced by a maximum of 64% and 68%, respectively. This effect was maintained through week 16, with mean reductions of 41% to 55% for TG and of 42% to 53% for VLDL-C [86]. The next step was to evaluate escalating doses (10, 25, 50, or 100 mg) of ARO-APOC3, given on days 1 and 29, to patients with TG > 300 mg/dL, whereas the only dose of 50 mg was given to FCS patients. When given to patients with high TG, ARO-APOC3 reduced apoC-III levels by 80% with the dose of 10 mg, by 98% with the doses of 25 mg and 50 mg, and by 99% with the dose of 100 mg. In FCS patients, the reduction of apoC-III levels was 99% upon administration of 50 mg ARO-APOC3. No clinically significant adverse changes were found in platelets, total bilirubin, or creatinine. ALT elevations were generally transient [87]. The next achievement has been the completion of the enrolment, on March 2023, of subjects for the phase III PALISADE trial to treat FCS (NCT05089084). The primary endpoint of this trial is the percent change from baseline in fasting TG at month 10. A total of 75 subjects receive 25-mg ARO-APOC3, 50-mg ARO-APOC3, or matching placebo once every 3 months. Participants who complete the randomized period will be eligible to continue in a 2-part extension period, when all participants will receive ARO-APOC3 (Table 2).

**Table 2:** Clinical trials testing safety and efficacy of ARO-APOC3 and ARO-ANG3

Trial identifier	Design	Population
ARO-APOC3		
NCT03783377 or AROAPOC31001	Phase 1	1) Healthy volunteers with triglycerides > 80 mg/dL 2) Patients with severe hypertriglyceridemia 3) Patients with familial chylomicronemia syndrome
NCT04720534 or AROAPOC3-2001 or SHASTA-2	Phase 2b	Individuals with severe hypertriglyceridemia (fasting triglycerides ≥ 500 mg/dL at screening)
NCT04998201 or AROAPOC3-2002 or MUIR	Phase 2	Patients with mixed dyslipidemia (mean fasting triglycerides ≥ 150 mg/dL and ≤ 499 mg/dL during screening)
NCT05089084 or AROAPOC3-3001 or PALISADE	Phase 3	Patients with familial chylomicronemia syndrome
ARO-ANG3		
NCT03747224 or AROANG1001	Phase 1	1) Healthy volunteers 2) Dyslipidemic patients (including heterozygous familial hypercholesterolemia and severe hypertriglyceridemia) 3) Hepatic steatosis (liver fat content > 10%)
NCT04832971 or AROANG3-2001 or ARCHES-2	Phase 2	Adults with mixed dyslipidemia
NCT05217667 or AROANG3-2003 or Gateway	Phase 2	Homozygous familial hypercholesterolemia

Other two phase 2 studies are the SHASTA-2 (Study to Evaluate ARO-APOC3 in Adults With Severe Hypertriglyceridemia) trial enrolling patients with severe hypertriglyceridemia (NCT04720534) and the MUIR (Study of ARO-APOC3 in Adults With Mixed Dyslipidemia) trial on patients with mixed dyslipidemia. This latter is active but not recruiting (NCT04998201) (Table 2).

#### Safety

No serious or severe adverse events were reported. In the AROAPOC31001 study, one patient receiving ARO-APOC3 experienced moderate transient ALT elevation with a peak of 210 U/L. However, this individual already had elevated ALT at baseline (65 U/L), with a return to baseline (45 U/L) by end-of-study (day 113). The rate of local injection site reactions was more common at higher doses in patients receiving ARO-APOC3 [86].

### ANGPTL3

ANGPTL3 is a 70kDa protein mainly expressed and secreted by the liver which is involved in the regulation of breakdown and lipid storage. ANGPTL3 decreases the clearance of very low-density lipoprotein-TG by inhibiting lipoprotein lipase activity and by a direct activation of lipolysis in adipocytes [88].

ARO-ANG3 is a RNA interference therapy targeting hepatic *ANGPTL3* mRNA. In ARO-ANG3, each RNA strand is 2′-methoxy (or 2′-fluoro) and phosphorothioate modified to induce resistance to endonucleases and offset immune activation. The sense strand additionally contains two inverted abasic subunits and an N-acetylgalactosamine targeting moiety [89]. This RNA-based approach has been tested in a phase 1 trial (NCT03747224) with the aim to assess safety and pharmacodynamic of single and multiple doses in four cohorts of 52 healthy individuals (TG > 100 mg/dL and LDL-C > 100 mg/dL) and one cohort of 9 healthy participants with hepatic steatosis (liver fat content > 10%). Healthy participants assigned to the single ascending dose arm received 35 mg, 100 mg, 200 mg, or 300 mg ARO-ANG3 or placebo subcutaneously on day 1. In the repeat dose design (not placebo controlled), individuals received 100-mg, 200-mg, and 300-mg ARO-ANG3 on days 1 and 29. Patients with hepatic steatosis were given 200 mg ARO-ANG3 on days 1 and 29. Dose-dependent reductions in ANGPTL3 were found in the overall cohorts. Specifically, in the single ascending dose group, mean changes from baseline at day 85 ranged from −44.7 (35 mg) to −77.8% (300 mg), an effect which was maintained in the cohort randomized to multiple ascending doses (changes from baseline at day 113 ranged from −64.4 (100 mg) to −92.7% (300 mg)). In subjects with hepatic steatosis, ANGPTL3 plumed 85.3% from baseline at day 113. Relative to TG, median percentage changes from baseline to day 85 ranged from −16.6 (35 mg) to −54.4% (300 mg); concerning non-HDL-C, changes at day 85 ranged from −28.7 (100 mg) to −17.5% (200 mg). TG and non-HDL-C were significantly reduced also in the case of multiple dose design and in individuals with hepatic steatosis [89].

A placebo-controlled phase 2b trial, named ARCHES-2 (Study of ARO-ANG3 in Adults With Mixed Dyslipidemia) is ongoing with the purpose to test the efficacy and safety of ARO-ANG3 in participants with mixed dyslipidemia (Table 2).

Since the inhibition of ANGPTL3 by the monoclonal antibody evinacumab led to a consistent reduction of LDL-C in HoFH patients [90], ARO-ANG3 has been initially tested in HeFH patients and in non-FH patients with LDL-C > 70 mg/dL despite statins. In HeFH patients, ARO-ANG3 (at the doses of 100, 200, and 300 mg) significantly reduced, in a dose-dependent manner, ANGPTL3 levels between 62 and 92% at week 16. LDL-C and TG were reduced in the range between −23 and −37% and between −25% and −43%, respectively. In non-FH patients, ARO-ANG3 (200 mg) reduced ANGPTL3 by 85%, LDL-C by 28%, and TG by 29% [91]. ARO-ANG3 will be tested also in HoFH patients enrolled in the Gateway (Study of ARO-ANG3 in Participants With Homozygous Familial Hypercholesterolemia) trial (NCT05217667). Patients will receive 2 open-label doses of ARO-ANG3 and be evaluated for safety and efficacy through 36 weeks. Those who will complete the first 36 weeks of treatment may opt to continue in an additional 24-month extension period during which they will receive up to 8 open-label doses of ARO-ANG3 (Table 2). Finally, although not in the remit of the of the present review, it is important to recall that another RNA-based approach against ANGPTL3 has been halted in early 2022 due to dose-dependent increases in liver fat and elevations in the liver enzymes ALT and AST at higher doses. Vupanorsen is a GalNac conjugated antisense oligonucleotide targeting ANGPTL3 which was tested in adults with non-HDL-C ≥100 mg/dL and TG between 150 and 500 mg/dL. Although the primary endpoint, namely the reduction of non-HDL-C, was met, elevations in ALT or AST >3× the upper limit of normal were more common with vupanorsen than placebo. The hepatic fat fraction increased with vupanorsen up to a 76% relative increase compared with baseline at the higher doses [92].

#### Safety

ARO-ANG3 was generally well tolerated with no apparent adverse effects on liver transaminases. When transient mild elevations in ALT were observed with ARO-ANG3, these cases were associated with use of a concomitant hepatotoxic supplement or medications and were self-limited. One participant receiving ARO-ANG3 demonstrated a post-dose peak increase in ALT >3× ULN, which was transitory. No thrombocytopenia, liver toxicity or changes in liver fat were observed. Mild injection site reactions were the most frequently reported TEAEs [89].

### Drugs in Early Development

Two siRNAs reducing ANGPTL3 levels have to be listed, namely, LY3561774 and ANGsiR10. The former is being tested in terms of safety, tolerability, pharmacokinetics, and pharmacodynamics in a phase 1 trial (NCT04644809) enrolling participant with dyslipidemia, whereas ANGsiR10 was studied only in mice and monkeys [93]. Relative to ANGsiR10, the siRNA sequences were designed to completely match with ANGPTL3 mRNA transcripts avoiding the recognition of the highly homologous genes such *ANGPTL4* and *ANGPTL8*, thus erasing potential off-target effects [94].

## Conclusions

**Fig. 2:**
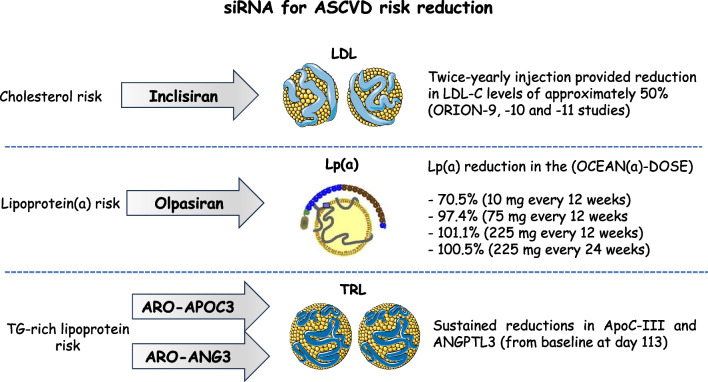
siRNA to reduce atherogenic lipoproteins. Inclisiran to lower LDL-C; olpasiran to lower lipoprotein(a); ARO-APOC3 and ARO-ANG3 to lower triglyceride-rich lipoproteins. ASCVD, atherosclerotic cardiovascular disease; LDL-C, low-density lipoprotein cholesterol; Lp(a), lipoprotein(a); TG, triglycerides; TRL, triglyceride-rich lipoprotein VLDL-C, very low-density lipoprotein cholesterol. (Parts of the figure were drawn by using pictures from Servier Medical Art. Servier Medical Art by Servier is licensed under a Creative Commons Attribution 3.0 Unported License https://creativecommons.org/licenses/by/3.0/)

In the context of current and future approaches to handle dyslipoproteinemias, the reviewed biosynthetic drugs hold promises for further improvements in the foreseeable future (Fig. 2). Significant progress has been made in drug development using RNA-based therapies aimed at treating difficult to target lipid disorders. Considering that duration of lipid-lowering effect is essential to achieve a greater cardiovascular benefit, siRNAs can improve patients’ adherence due to their ability to be administered every few months. In the era in which many patients require a combination of lipid-lowering agents to achieve the goals advocated by guidelines, the ability to introduce a further choice in clinical practice becomes an important step forward for patients [95].
